# Do Chief Executives Matter in Corporate Financial and Social Responsibility Performance Nexus? A dynamic Model Analysis of Chinese Firms

**DOI:** 10.3389/fpsyg.2022.897444

**Published:** 2022-05-27

**Authors:** R. M. Ammar Zahid, Muzammil Khurshid, Wajid Khan

**Affiliations:** ^1^School of Accounting, Yunnan Technology and Business University, Kunming, China; ^2^Department of Banking and Finance, University of the Punjab, Gujranwala Campus, Gujranawala, Pakistan; ^3^Department of Business Management, University of Baltistan, Skardu, Pakistan

**Keywords:** corporate social responsibility, corporate financial performance, CEO turnover, CEO-duality, dynamic model

## Abstract

This study examined the relationship between Corporate Financial Performance (CFP) and Corporate Social Responsibility Performance (CSRP). Furthermore, it explored the effectiveness of chief executive characteristics as a moderator in the CFP-CSRP nexus. We employed a dynamic sysGMM regression model on 2,439 firm-year observations of Chinese firms. The results reveal that CFP (market-based) has a significant positive impact on CSRP. However, CFP (historical) is significantly negatively related to CSRP. Furthermore, the study found that CEO turnover and CEO duality negatively moderate the CFP-CSRP relationship, while CEO as CFO positively moderates this relationship. The findings have substantial implications for all stakeholders, including investors, CEOs, corporate regulators, and policymakers.

## Introduction

In the last three decades, corporate social responsibility performance (CSRP) and corporate financial performance (CFP) have remained in academic discussion. Especially current digitalization revitalized the discussions (Rauf et al., 2020). CSR is the continuous legal commitment of a corporation toward the community, and there is a direct relationship between meeting CSR activities and shareholder wealth maximization (Safarzad et al., 2016). The primary reason for the CSR initiative by public and private companies is that CSR firms outperform non-CSR firms. However, the problem with CSR reports is that they are subjective and have inherited perceptual biases (Carroll, 1991). The main question in CFP and CSRP debate is whether socially responsible firms report the same financial performance compared to firms not involved in social investments? (Mcwilliams and Siegel, 2001; Choi et al., 2010; Rauf et al., 2020). The literature provides inconclusive evidence; the results of some studies are insignificant, some are negative or neutral (Moore, 2001), and a majority are positive impact studies (Kempf and Osthoff, 2007; Choi et al., 2010).

To be socially responsible and remain financially sustainable are two challenges faced by corporate at the same time. CSR ensures firm performance while protecting the social welfare of stakeholders (Lin et al., 2009), and socially responsible firms attract more investors. In this study, the CFP and CSR relationship was investigated in the presence of CEO turnover, CEO duality, and CEO as CFO. CEO is the leading player and is responsible for planning, organizing, controlling, and evaluating short- and long-term strategies. With CEO turnover or the arrival of new CEO, the overall affairs of the corporation are affected (Murphy and Zimmerman, 1993). How does the Chinese firm feel about this change? We found a significant negative impact of CEO turnover on CFP-CSR. CEO duality and CSR are essential aspects of corporate affairs. CEO duality may lead to concentration of power and less interest in CSR practices because CEO duality enables the CEO to exercise excessive decision-making power (Jensen, 1993). Removal of poor-performing CEO becomes challenging for the board when he holds the position of chairman (Goyal and Park, 2002). It is expected that the CEO duality may bring a problem to the governance and also a negative impact on CSRP. For significant internal control, the role of the CFO in top management is increased. As CEOs and CFO play different roles within corporations, their approach toward CSR is different. Our findings contribute to the existing financial reporting and management literature by measuring the CEO as CFO in the Chinese context. Our findings confirm that holding both positions by CEO increases the CSR performance of Chinese firms.

This study is a tangible contribution to the literature on corporate financial and CSR performance and chief executives' role in this nexus. Previous literature provided inconclusive evidence on the CFP and CSRP relationship. Further, there are studies investigating the direct impact of CEO and CSR performance. To the best of our knowledge, there is no study investigating the moderating role of chief executives in CFP-CSRP nexuses. Therefore, this study fills this gap by investigating how particular characteristics of CEOs (i.e., CEO duality, CEO turnover, and CEO as CFO) influence the CFP-CSRP relationship. CFP performance plays a diverse role in a firm's CSR performance in China, and minimal literature is available on this issue in the Chinese context, especially from the chief executives' perspective. By focusing on the equity market of China, this study is a unique contribution to the existing literature in the following ways. First, most corporations in China are state-owned; the state sets all the relegations as the actual owner. It allows the government to control corporations and set policies in the best interest of the state and all stakeholders. In this unique scenario, the role of chief executives is very important. Like other emerging economies in the world, the Chinese corporations are subjected to following state rules and regulations, dividend disbursements, and maintaining a competitive edge in the equity market. Second, CSR reporting under the article of the Shanghai and Shenzhen Stock exchange memorandum obliged the firm to maintain audited reports on environmental, economic, and social aspects. Third, the moderating role of CEO turnover, CEO duality, and CEO as CFO in CFP and CSRP nexus is rarely examined in the existing literature. Executives play a vital role to settle the conflict between executives and shareholders. That is considered the root cause of agency problems. To better understand this relationship and mitigate its potential impact, we select CEO turnover, CEO duality, and CEO as CFO as moderating variables between the CFP and CSRP. It helped us understand if the CEOs impact the CFP-CSRP nexus.

The rest of the paper is organized as follows: Section two explains various CSR theories. Section three explains the sample and research methodology. Section four is about results and analysis. Section five concludes and adds policy implications for various stakeholders.

## Theoretical Background and Hypothesis Development

Various theories are used in the literature to relate corporate financial and sustainability performance, such as stakeholder theory (Van Der Laan et al., 2008), legitimacy theory (Nicholls, 2010), institutional theory (Collin et al., 2009), and signaling theory (Connelly et al., 2011).

The stakeholder theory is considered the most applied theory because there are wider stakeholders in firms than shareholders and investors (Safarzad et al., 2016). Carroll (1991) identified four parts of CSR that society expects from corporations: economic, legal, ethical, and philanthropic. His emphasis is that all CSR obligations should be achieved logically, first economic, followed by philanthropic. Freeman (2010) examined the relationship between stakeholder theory and CSR. Freeman's theory proposed that a corporation can only achieve sustainable goals by protecting the interest of all its stakeholders. The term stakeholder includes a person or group who has a claim, right, or interest in a corporation and its present, past, and future. Who can be affected is an essential aspect of stakeholder theory. Ultimately, they also affect the corporation's affairs (Clarkson, 1995). The theory explains that responsible corporations toward society have better financial performance (Freeman, 2010). The theory further elaborates on “secondary stakeholder” and its relationship with the corporation. Secondary stakeholder includes community activist, civil society organization, and social moments (Russo and Perrini, 2010). There are discussions and agreements that these stakeholders have no legal claims on corporation affairs, so they should not be treated as stakeholders (Clarkson, 1995; Russo and Perrini, 2010; Khan et al., 2021). According to the legitimacy theory, companies operate under a “social agreement” that strives to win and maintain social recognition that supports understanding the need for businesses to produce sustainability reporting practices that attempt to defend the legality of their operations. The legitimacy method explains why sustainability reporting has become a “moral” need (Nicholls, 2010; Khan et al., 2022).

The stakeholder theory, which is particularly valuable for justifying and assessing the causes of non-financial reporting's sustainability, is inextricably tied to the legitimacy theory. The stakeholder theory explains how a business interacts with external and internal stakeholders. It shows why stakeholders' information requirements for sustainability reporting entail a multidimensional display of economic, social, and environmental potential. According to the stakeholder theory, these implications can be evaluated in three ways: first, the descriptive element, which assesses a firm's reporting behavior while taking into consideration the competing interests of the organization and its stakeholders. Second, there is the operational side, which is concerned with achieving organizational goals and presenting those accomplishments through reporting. Third, the normative aspect aids in analyzing conformance with norms and rules based on moral principles, given that stakeholders have the power to affect the business and contribute significant value to the organization. It also includes rules and recommendations, bringing the stakeholder theory closer to the normative accounting theory (Donaldson and Preston, 1995).

The institutional theory examines the institutionalization of social characteristics and can be used to understand better how a business entity interacts with its stakeholders. According to institutional theory, organizations are influenced by their institutional surroundings, consisting of socially constructed norms and rationales. These laws govern organizational behavior and activities. As a result, the idea raises awareness of a new institutional space, implying that socially responsible firms must consider economic, environmental, and social factors (Collin et al., 2009). Non-financial reporting also reflects the need to reduce information asymmetry between internal and external stakeholders. The construction of an “information bridge” between stakeholders is the core concept of the signaling theory.

We conclude the discussion of CSR theories with the idea that all of them aid in a better understanding of non-financial reporting's evolution to assuage concerns about managers abusing their information advantage. Further, all theories stress the financial obligation of corporations to perform their operation legally, economically, and philanthropically. Complete transparency from all stakeholders must be required on the implementation side. Lack of transparency is the leading cause of exploitation and further leads to the agent principle problem or agency theory proposed by Jensen and Meckling (1976). All socially responsible enterprise model participants should benefit from managerial “information signals.” This essential premise explains the scope of sustainability reporting. Finally, these theories will serve as a theoretical foundation for understanding CSR and corporate performance literature. The theories claim, in particular, that adopting social and environmental practices allows businesses to save money on production expenses by lowering environmental hazards while simultaneously enhancing relationships with key stakeholders. It will help the company gain a competitive advantage and, as a result, improve its long-term financial success.

### Corporate Financial Performance and CSR Performance Nexus

Transformation in executive positions occurs in every organization. They are of various kinds such as, a term of the specific year is completed, in the form of retirement, force terminations, and organizational changes in the organizations' top levels for the new venture. The Chinese equity market is still in the developing stage; replacing traditional CEO with new talent will effectively turn corporations' poor financial positions. Executives of longer terms in the developing world have certain disadvantages. First, executives develop relationships with board members and dominate the corporation's strategic decisions. Second, there is a potential conflict of interest between executives and shareholders, and corporate governance is a mechanism used to deal with this conflict, decreasing the CFP of a firm (Morellec et al., 2012).

CEO departures are either by choice or force. The retired CEO leave a legacy and potential problems which negatively affect firm performance (Dikolli et al., 2014). As usual, corporations continue their operation become problematic because they have been policymakers for years, and hence their replacement for corporations is a challenging task. The CEOs who retired at their times and firm performance remained sustainable and better received lucrative retirements benefits (Murphy and Zimmerman, 1993). The continuous issuance of CSR reports helps firms be considered business-friendly and positively affects investors' attitude toward executives despite involvement in misconduct (Christensen, 2016; Khan et al., 2021).

Entrenchment theory proposes that CEOs use CSR for job protection and to reduce CEO turnover. CEOs lead corporate employees to engage in CSR activities. CEO turnover is an integral part of corporate culture and governance (Kor, 2006). With the departure of the old CEO and the arrival of the new CEO, there will be more board meetings, and the board of directors will be careful in decisions because of the high frequency of meetings, and the newly appointed CEO will make new decisions. One disadvantage associated with the new CEO is that the new management's earnings tend to decrease to blame the former management and show a significant rebound next year (Angelo, 1988).

On the other hand, the new CEO will tend to increase investment in R&D on purpose to increase profit. In all these battles of interest, executives play a negative role in CSR reporting (Rauf et al., 2021). Executive turnover occurs for many reasons, but its impact on CSR is complicated. From the discussion, we can conclude that executive turnover negatively moderates the relationship between corporate financial performance and CSR performance. So, we develop the following hypothesis:

**H_2_**: *The CEO turnover is negatively moderating in the CFP-CSRP relationship*.

More than half of S&P 500 companies' CEOs hold two posts (i.e., merging the chairman of the board and CEO of the firm), yet corporate stakeholders consistently urge enterprises to maintain these two positions distinct (Goergen et al., 2020). According to agency theory, duality strengthens the CEO's authority, and CEOs' private interests are likely to influence their participation in CSR initiatives. It affects the decision-making by compromising necessary independence, which may negatively affect corporate performance (Fama and Jensen, 1983). Further, It also negatively affects the monitoring of businesses (Tuggle et al., 2010). In comparison, others suggest that CEO duality provides an adaptive and focused leadership with timely decisions that help improve organizational performance unpredictably (Dahya et al., 1996). According to the upper echelons theory, CEOs influence CSR action through their attitudes and values. When analyzing business responses to external challenges, executives' relevant attributes are crucial determinants (Lewis et al., 2014; Zahid and Simga-Mugan, 2019). CEOs choose self-interest when sharing environmental data, lowering CSR quality (Meng et al., 2013). While separating the chair and CEO, duties will increase sustainably (Naciti, 2019). The CEO duality significantly reduces board meetings and strategic planning if CEO also works as chairman of the board. In corporate governance, it is called share/chair duality. Corporation strategic decision-making and implantation are controlled and directed by top managers in a more timely fashion (Rauf et al., 2021).

The above discussion and Chinese board structures suggest that CEOs may direct the firm according to the communist party charter or personal objectives by exercising their dual office power and controls. So, we hypothesize that CEO duality may negatively moderate the CFP-CSRP relationship.

***H_3_:*** *The CEO duality is negatively associated with the CFP-CSRP relationship*.

CFOs are mainly responsible for the investment and financing decisions of the corporations. BESIDES, the CFO considers a prominent CEO partner and assists and reports various financial results to company investors and policymakers. Kuehn (2010) reported that the CFO's responsibilities are to drive the accounting and report and to be responsible for economic, social, and environmental issues. This dual role CFOs can be classified as focusing on financial performance and corporate social responsibility. The role of the CFO becomes informant increasingly after the corporate scandals of WorldCom and Enron in the early 2000s and the implementation of legislation (Sarbanes-Oxley act) that increase the accountability of the decision-makers.

Interestingly, the CEO still holds the power of significant investment and financing decisions. Moreover, CFOs assist in financial and strategic decision-making (Tulimieri and Banai, 2010). The CFO and the CEO communicate to firm investors and strategic analysts (Tulimieri and Banai, 2010). Many CFOs reported manipulated earnings due to pressure from the CEO (Feng et al., 2011), resulting in the resignations of CFOs because of income-increasing accruals demands from the CEO's office. An exciting phenomenon occurs when one person holds both positions; on one side, it seems to increase in work burden on the CEO. However, on the other hand, there is a high probability of accurate financial reporting to various stakeholders that may increase the firm's confidence and overall performance (Zahid et al., 2018). In this study, we include CEO as CFO to examine whether holding both positions by one person has any significant impact on corporate financial performance and the CSRP relationship. From the above discussion, we can develop the following hypothesis:

**H_4_:** *CEO as CFO is positively associated with the CFP-CSRP relationship*.

## Data and Methodology

### Data Description

To meet our objectives, data were collected from the Chinese Stock Market and Accounting Research (CSMAR) database for the period 2009–2016. Initial data included listed firms on Shenzhen and Shanghai stock exchanges; we excluded the financial firms from the dataset and included firms that published CSR reports observations. After deleting the missing values, the final sample consists of 2,439 firm-year data estimated to draw inferences about the study variables.

One of the main problems with the Chinese firms' CSRP and CEO data is their cross-section availability. However, a detailed analysis should cover the longitudinal data to uncover fundamental aspects of performance (Haque and Ntim, 2018; Khan et al., 2021). Therefore, this study combines the cross-sectional and longitudinal data to develop a panel time-series database for analysis and cognizant conclusions. Since the current CSR performance is also affected by the last CSR activities, the current research examines the CFP–CSRP relationship in a dynamic framework. Further, the presence of possible correlations between the endogenous variables such as corporate performance, CEO characteristics, and other control variables also motivate to use a dynamic model. We mainly employed the sysGMM estimation approach to analyze the CFP-CSRP relationship in a dynamic framework. As the sysGMM account for the lagged values, the variables with the missing values in three consecutive years have been deleted.

### Dynamic Research Model

The following sysGMM model was developed to estimate the relationship between CSR performance and corporate financial performance for firm i in the time period t:


(1) $$\begin{array}{l} CSRP_{it}=CFP_{it}\beta_{CFP}+\;C_{it}\beta_{C}\;+\;X_{it}\partial +\;Z_{it}\beta_{Z}\\ \;+\rho CSRP_{i(t-1)}+\varsigma_{i}+\in_{it} \end{array}$$

Where

- **CSRP**_**it**_ is a firm of Corporate Social Responsibility Performance measured by CSR scores.
-**CFP**_**it**_ is a vector of Corporate Financial Performance measured by Tobin's Q and ROA alternatively.
-**C**_**it**_ is a vector to control governance-related effects measured by Board Size.
-**X**_*it*_ represents financial performance-related control variables such as the size of the firm, financial leverage, and total assets, which may affect the CFP-CSRP relationship.
-**Z**_**it**_ represents the pairwise interaction among CFP, C, and X elements.
-**ς**_**i**_ is firm non-observable heterogeneity.
-**∈**_**it**_ is the specific error term.
-**i** represents firm *i*, and ***t*** fiscal year *t*.
-While β_CFP_, β_C_, β_z_, and α are comparable parameter vectors.

The model assumes AR (1) in firm CSRP with first-order correlation p.

There is the possibility of complication while estimating equation (1) because independent variables may correlate with unobserved firm heterogeneity factor Ci and affect past CSR performance (dynamic endogeneity). Therefore, Pooled OLS and Penal Fixed Effect are inconsistent and skewed. The System Generalized Method of Estimation was developed by Arellano and Bover (1995) and Blundell and Bond (1998). By using sysGMM, the piled system of level and the first difference can be estimated in the following equation in our case,


(2) $$\begin{array}{l} CSRP_{it}=CFP_{it}\beta_{CFP}+\;C_{it}\beta_{C}\;+\;Z_{it}\beta_{Z}+\;X_{it}\partial \\ \;+\rho CSRP_{it-1}+\varsigma_{i}+\in_{it}CSRP_{it}CFP_{it}\beta_{CSRPCFP}\\ \;+C_{it}\beta_{C}+Z_{Iit}\beta_{Z}+X_{it}\partial +\rho CSRP_{i(t-1)}\\ \;+CSRP_{it}+\in_{it} \end{array}$$

Equation (2) includes a one-period performance lag and assumes it is sufficient for dynamic completion for exposition. Instruments included are thus ideal for lags >1.

The following relation at level and first difference instruments sets are,


(3) $$\begin{array}{l} (\Delta CFP_{i\left(t-2\right)},\ldots ,\Delta CFP_{i\left[t-\left(t-2\right)\right]},\;\Delta C_{i\left(t-2\right)},\ldots ,\Delta C_{i\left[t-\left(t-2\right)\right]},\;\\ \Delta Z_{i\left(t-2\right)},\ldots ,\Delta Z_{i\left[t-\left(t-2\right)\right]}\;,\Delta X_{i\left(t-2\right)},\ldots ,\Delta X_{i\left[t-\left(t-2\right)\right]}\\ \left(CG_{i(t-2)},\ldots ,CG_{i\left[t-\left(t-2\right)\right]},\;Z_{i\left(t-2\right)},\ldots ,Z_{i\left[t-\left(t-2\right)\right]}\;,X_{i\left[t-\left(t-2\right)\right]})\right) \end{array}$$

In the case of unbalanced Panel data difference formulation in equation (2), data losses with sample gaps can result in data losses. **CSRP**_**it**_ followed by **CSRP**_**i**(**t**−**1**)_ will be missing if **CSRP**_**it**_ is missing. The forward orthogonal deviation proposed by Arellano and Bover (1995) is the alternative to the first difference equation in response to sample gaps and data missing problems. In this technique, instead of taking simple lag or first difference, the average of all expected future values of the respective variables is subtracted from the current value of that variable. In the calculation process, only the last variable value is missed. Even if there are gaps, it works well. Further, lag values can be used as instrument variables as there is no interruption in forwarding deviations.

The following model has been developed to analyze the moderating role of CEO characteristics in the CFP-CSRP nexus.


(4) $$\begin{array}{l} CSRP_{it}=CFP_{it}\beta_{CFP}+CEO_{it}\beta_{CEO}\\ \;+CEO\#CFP_{it}\beta_{CEO\#CFP}+\;C_{it}\beta_{C}\;+\;X_{it}\partial \\ \;+Z_{it}\beta_{Z}+\rho CSRP_{i(t-1)}+\varsigma_{i}+\in_{it} \end{array}$$

Where

-**CEO** represents the CEO turnover, CEO duality, and CEO role as CFO alternatively.
-**CEO#CFP**_**it**_ shows the interaction term to account for the moderating effect of CEO characteristics in the CFP-CSRP nexus.
-The rest of the variables, such as CSRP, CFP, C, X, Z, and so on, represent the same variables as Equation 1.

Overall, the sysGMM estimation technique has several advantages over its counterparts, that is, simple/pooled OLS estimation or fixed/random effects estimation techniques. It incorporates the firm's fixed effect to tackle unobserved heterogeneity in its variables data. Further, it works in a dynamic environment by including the past values of CSR performance and other model variables. It provides robust results by removing the firm's level heteroscedastic and serial correlations issue. It also works well in unbalanced panel data with gaps, as is the case in this study. Finally, sysGMM incorporates the set of internal instrument variables such as lagged and differences values of CSR, C, Z, X, and firm performance to account for endogeneity issues of data, so there is no need to search for any external instrument variables.

### Variable Description and Measurements

#### Dependent Variable

The dependent variable “CSR performance” is measured with a reporting index developed on listed firms' various CSR reporting quality parameters based on previous work (Khan et al., 2013; Sial et al., 2018; Rauf et al., 2020). The complete items included in the CSR reporting index are given below in Table 1.

**Table 1:** CSR reporting index.

**S. No**	**CSR reporting**	**Scale**
1	GRI sustainability guidelines reported or not	1, 0
2	Shareholder interest protection reported or not	1, 0
3	Creditor interest protection reported or not	1, 0
4	Environment and sustainability are reported or not	1, 0
5	Supplier's interest protection reported or not	1, 0
6	Consumer and clients interest protection reported or not	1, 0
7	Employee interest protection reported or not	1, 0
8	Secure production reported or not	1, 0
9	Social responsibility system construction and reporting are reported or not	1, 0
10	Public relations, social, and public welfare are reported or not	1, 0
11	Deficiency of company reported or not	1, 0

The CSR rating is calculated by $\Sigma_{n}^{1}\left[\left(x/n*100\right)\right]$., where n represents all items and x = 1 if an item is reported and 0 otherwise.

The CSR rating is calculated by $\overset{1}{\underset{n}{\sum }}{\frac{x}{n}}^{*}100$, where n represents all items and *x* = 1 if an item is reported and 0 otherwise.

#### Independent Variables

The independent variable for this study is Corporate Financial Performance (CFP). In the literature, the CFP is measured through different proxies. In this study, we used two measures, one is Tobin Q and the other is Return on Assets (ROA). Both of them measure different perspectives of financial performance. Tobin's Q is the wildly used measure of financial performance to the prospects of return, keeping the firm physical value. At the same time, ROA is historic and represents a corporation's past financial performance.

Tobin's Q is a market-based performance measure that anticipates the investors' future worth and returns of firms. On the other hand, Return on Assets (ROA) is based on historical financial performance measured by the net income generated from operating, financing, and investing activities divided by the total assets (Mueller and Peev, 2007). Comparatively, changes in accounting regulation and treatments significantly impact accounting-based financial performance, whereas investors' sentiments and expectations have less impact. Overall, market-based measures, that is, Tobin's Q, incorporate additional information from the anticipations of investors and relatively gets faster reflections (Demsetz and Villalonga, 2001).

#### Moderating and Control Variables

CEO turnover, CEO duality, and CEO-CFO are used as moderating variables to measure the impact of different roles of the CEO in the CFP-CSRP relationship. Further, control variables are selected based on previous studies like board size, firm size (Rauf et al., 2020), firm age (Cumming et al., 2016), and financial leverage (Rauf et al., 2021). All variable measurements are given in Table 2.

**Table 2:** Variable measurement and descriptive statistics.

**Variable**	**Measurement**	**Obs**	**Mean (SD)**	**Min–Max**
CSRP	The CSR Performance is calculated by the formula $\overset{1}{\underset{n}{\sum }}\frac{x}{n}\;*\;100$, where n represents all items and *x* = 1 if an item is reported and 0 otherwise (Details Table 1)	4,241	36.39 (15.233)	0–89.3
Board size	The total director mentioned on the company board	4,193	9.519 (2.301)	4–22
CEO turnover	We measure CEO turnover by giving a value 1 if CEO turnover occurred in the current year and 0 otherwise	4,198	0.552 (0.497)	0–1
CEO duality	1 if CEO is also a chairman otherwise 0	4,142	0.154 (0.361)	0–1
CEO–CFO	1 if CEO is also a CFO of company; otherwise 0	4,198	0.348 (0.476)	0–1
Tobin's Q	The percentage of the market plus additional physical value	3,755	1.764 (1.839)	0.097–33.27
ROA	Return on assets = (Net profit/Total assets)	3,841	0.043 (0.058)	−0.691–0.482
ROE	Return on equity = (Net profit/Total equity)	3,841	0.086 (0.798)	−15.80–43.61
Age (ln)	Natural log of the number of years since the firm is listed	4,237	2.686 (0.437)	0–3.611
Size	Natural log of the total assets of firm	4,199	23.209 (1.768)	18.27–30.81
Sale (ln)	Revenue in RMB	3,716	22.455 (1.664)	17.43–28.66
BTM	Book value to market vale = (TotalEquity/market capitalization)	3,755	1.178 (1.135)	0.03–10.329
Financial leverage	FL = total debt/total assets	4,199	0.519 (0.213)	0.009–1.51

## Results

### Descriptive Statistics

Descriptive statistics are reported in Table 2. The CSR rating mean value is 36.39 and has a Standard deviation of 15.233. It indicates that the Chinese firms, on average, accomplish 36.39 composites with the highest rating of 89.3. The average board size is 22 and the average number of members on board is 9. The executive turnover show 5% in the Chinese-selected firms. CEO duality figures show that in <2% of the firm, both chairman and CEO responsibility is shouldered by one person. The financial performance measures indicate that Tobin's Q is on the higher side than ROA and ROE, which means that expected returns are higher than historical returns.

### Strict Exogeneity Tests

It is essential to check the homogeneity of variables used in the dynamic model before applying the sysGMM. Thus, we applied a strict exogeneity test in the panels (T > 2) as defined by Wooldridge (2010). Precisely, the following equation was estimated:


(5) $$\begin{array}{l} CSRP_{it}=CFP_{it}\beta_{CFP}+\;C_{it}\beta_{C}\;+\;X_{it}\partial \\ \;+\;Z_{it}\beta_{Z}+\delta W_{i(t-1)}+\varsigma_{i}+\in_{it} \end{array}$$

Where

-**W_i(t−1)_** is a vector of one-period lead values of CFP and other model variables (i.e., C, Z, and X).
-The rest of the variables, such as CSRP, CFP, C, X, Z, and so on, represent the same variables as given in Equation 1.

To test the strict exogeneity, **δ** of *W*_*i*_ has been analyzed by estimating Equation 4 (**δ** = 0, represents the strict exogeneity). Further, we used the industry and year-fixed effects with robust standard errors. Table 3 shows the estimated results of **δ** for both financial performance measures (i.e., Tobin's Q and ROA).

**Table 3:** Strict exogeneity tests.

**Variables**	**CSRP**	**CSRP**
	**I**	**II**
Tobin's Q *(t + 1)*	0.291**	
	(0.0822)	
ROA *(t + 1)*		4.109*
		(0.899)
CEO turnover *(t + 1)*	−2.417***	−6.09**
	(0.653)	(1.891)
CEO duality *(t + 1)*	−0.270*	−0.199**
	(0.265)	(0.179)
CEO–CFO *(t + 1)*	−0.0344**	−0.242***
	(0.0135)	(0.0424)
Size (total assets) *(t + 1)*	−0.568***	−0.376*
	(0.0894)	(0.275)
Age *(t + 1)*	−0.570	−0.418
	(0.483)	(0.854)
Sales *(t + 1)*	−0.331	−0.732
	(0.287)	(0.948)
Financial leverage *(t + 1)*	0.706*	0.971***
	(0.299)	().341)
MTB (*t* + 1)	0.747	0.761***
	(0.352)	(0.312)

1. ***p <0.01, **p <0.05, *p <0.1, respectively.

*2. () parentheses show the robust standard errors.*

*3. Where CSRP represents corporate social responsibility performance. ROA, Returns on assets; MTB, Market to book value*.

In Table 3, columns 2 and 3 represent that CSR performance is not strictly exogenous with financial performance (Tobin's Q and ROA) and other moderating variables (CEO Turnover, CEO Duality, and CEF–CFO). Similarly, control variables of the study, that is, Size, Age, Sales, Financial Leverage, and MTB, are also not strictly exogenous with CSR performance. Overall, the results suggest that the strict exogeneity hypothesis cannot be rejected.

### CSR Performance-Corporate Financial Performance Nexus

Table 4 shows the covariates and estimates the impact of CFP on CSRP. Using the system GMM model, the results reveal that Tobin's Q has a significant positive impact on CSRP (0.340^***^). It indicates that with future expected financial performance, corporation tends to increase CSR performance, so it supports the hypothesis (H_1_). However, Return on Assets has a significant negative impact on CSR performance (−6.915^***^). The negative coefficient of ROA supports H_1a_ and entrenchment theory. The control variables, that is, size, sales, and board size, have a significant positive effect on CSRP. In contrast, Age, Financial Leverage, and Book to market value have a significant negative impact on CSRP. These findings align with previous literature (Rauf et al., 2021).

**Table 4:** CSRP–CFP relationship (system GMM parameter estimates).

**Variables**	**CSRP**	**CSRP**
	**I**	**II**
Tobin's Q	0.340***	
	(0.0794)	
ROA		−6.915***
		(2.416)
Size	2.373***	2.230***
	(0.194)	(0.193)
Sales	0.604***	0.728***
	(0.160)	(0.164)
Age	−1.822***	−1.910***
	(0.291)	(0.292)
Board size	0.234***	0.216***
	(0.0574)	(0.0574)
Financial leverage	−3.080***	−4.780***
	(0.782)	(0.815)
BTM	−0.945***	−1.104***
	(0.134)	(0.134)
CSRP (*t*−1)	0.492***	0.490***
	(0.00911)	(0.00923)
Constant	−42.53***	−39.61***
	(2.558)	(2.434)
Observations	2,439	2,439
Number of id	685	685

1. ***p <0.01, **p <0.05, *p <0.1, respectively.

*2. () parentheses show the robust standard errors.*

*Where CSRP represents corporate social responsibility performance. ROA, Returns on assets; MTB, Market to book value*.

### Moderating Effect of CEO Turnover, CEO Duality, and CEO as CFO on CSRP-CFP Nexus

Table 5 presents the moderating effect of CEO turnover (column 3, 4), CEO duality (column 5, 6), and CEO as CFO (column 7, 8) on the CSRP-CPF nexus. To capture the moderating effects, we developed the interaction term of CEO turnover, CEO duality, and CEO-CFO with each variable of CFP. Columns 3 and 4 of Table 5 show that the interaction term of CEO turnover and CFP (**CEO Turnover # Tobin's Q**) has a positive but nonsignificant impact on CSR performance. However, the interaction term (**CEO Turnover # ROA)** is significantly and negatively related to CSR performance. It represents that even if the CEO change in a firm with historical profitability, the CSR performance will be reduced. The results are consistent with Dikolli et al. (2014).

**Table 5:** Moderating effect of CEO role in CSRP–CFP Nexus (sysGMM parameter estimates).

	**CEO turnover**		**CEO duality**		**CEO–CFO**	
**Variables**	**CSRP**	**CSRP**	**CSRP**	**CSRP**	**CSRP**	**CSRP**
	**I**	**II**	**I**	**II**	**I**	**II**
Tobin's Q	0.0458		0.530***		0.396***	
	(0.193)		(0.138)		(0.148)	
ROA		−3.020		1.529		−9.095**
		(7.411)		(3.554)		(4.541)
CEO turnover	−2.498**	−2.154**				
	(1.153)	(1.044)				
CEO duality			−2.261	2.395		
			(1.974)	(2.112)		
CEO–CFO					2.398*	2.355**
					(1.376)	(1.060)
CEO turnover #	0.673					
Tobin's Q	(0.414)					
CEO turnover #		−6.740*				
ROA		(11.45)				
CEO duality #			−0.654*			
Tobin's Q			(0.563)			
CEO duality #				−82.17***		
ROA				(25.87)		
CEO–Cfo #					−0.177	
Tobin's Q					(0.436)	
CEO–CFO # ROA						5.980*
						(9.851)
Size	2.397***	2.262***	2.491***	2.204***	2.345***	2.199***
	(0.198)	(0.200)	(0.200)	(0.197)	(0.198)	(0.198)
Sales	0.574***	0.646***	0.520***	0.775***	0.593***	0.715***
	(0.165)	(0.171)	(0.163)	(0.169)	(0.162)	(0.167)
Age	−1.813***	−1.939***	−1.909***	−1.894***	−1.794***	−1.872***
	(0.297)	(0.301)	(0.293)	(0.296)	(0.296)	(0.299)
Board size	0.259***	0.255***	0.164***	0.188***	0.253***	0.234***
	(0.0603)	(0.0614)	(0.0626)	(0.0651)	(0.0590)	(0.0593)
Financial	−2.884***	−4.732***	−3.157***	−4.926***	−2.805***	−4.382***
leverage	(0.804)	(0.846)	(0.794)	(0.832)	(0.816)	(0.846)
BTM	−0.913***	−1.083***	−0.983***	−1.182***	−0.925***	−1.083***
	(0.137)	(0.138)	(0.139)	(0.138)	(0.136)	(0.137)
CSR rating (*t*−1)	0.492***	0.491***	0.492***	0.491***	0.492***	0.489***
	(0.0926)	(0.0958)	(0.0917)	(0.0932)	(0.0923)	(0.0944)
Constant	−41.54***	−37.71***	−42.20***	−39.83***	−42.85***	−39.86***
	(2.711)	(2.606)	(2.634)	(2.562)	(2.603)	(2.497)
Observations	2,439	2,439	2,409	2,409	2,439	2,439
Number of id	685	685	683	683	685	685

1. ***p <0.01, **p <0.05, *p <0.1, respectively.

*2. () parentheses show the robust standard errors.*

*Where CSRP represents corporate social responsibility performance. ROA, Returns on assets; MTB, Market to book value*.

Columns 5 and 6 of Table 5 depict that the interaction term of CEO duality and CFP (CEO Duality # Tobin's Q and CEO Duality # ROA) has a significant negative impact on CSR performance. It shows that if CEO is also a chairman, it will decrease the CSR performance of the company. It means that CEOs in the dual role are avoiding CSR costs and are not motivated to serve the interest of various stakeholders. The same findings were reported by Khan et al. (2013) and Sundarasen et al. (2016).

Columns 7 and 8 of Table 5 demonstrate that the interaction term of CEO as CFO and CFP (**CEO-CFO # Tobin's Q**) has a negative but non-significant impact on CSR performance. However, the interaction term (**CEO-CFO # ROA)** positively relates to CSR performance. It means that if the CEO holds the office of CFO, it will increase the CSR performance of the company.

## Conclusions And Policy Implementations

The study aimed to examine the association between CFP and CSRP with the CEO's role as moderator. Notably, we answered four questions to validate the stakeholder theory in the context of the listed Chinese firms and want to answer whether CFP is responsible for CSR practices or it is just a belief that all those firms who work for the interest of all stakeholders are more profitable as compared to those that work only for stockholders wealth maximization. The second question is, how does executive turnover play a role in slowing down CFP if any? Furthermore, was CSR activities remain regular with the arrival or departure of the CEO? Third, the chairman works as a bridge between the corporate board and shareholders. Shouldering this task by CEO will have any significance for Chinese corporations, or separation will be the ideal scenario? Forth, CFO is responsible for controlling, evaluating, and reporting the financial results mainly to CEO and external institutional investors. If the CEO also performs this role, what are the possible implications for Chinese corporations? Using the sysGMM, the results contribute in the following ways. Return on Assets (Historical CFP) has a significant negative impact and Tobin's Q (market-based CFP) has a significant positive impact on CSRP. CEO turnover and CEO duality negatively moderate the CFP-CSRP relationship, while CEO as CFO positively moderates this relationship. Findings have substantial implications for all stakeholders, including shareholders, CEOs, corporate regulators, and policymakers.

Our study has many policy implications. First, firms that engage in CSR initiatives outperform their rivals financially. It is an encouraging sign that firm spending on CSR yields excess returns on average. CSR initiatives, on one side, reflect the strong corporate governance practices as the top level is involved. Thus, the execution of the CSR plan is treated as an investment decision with upfront cash outflow for long-term returns. The Chinese firms are politically embedded. The results are very encouraging for policymakers such as investors, corporate practitioners, and local government as normative and progressive values can be met with socially responsible operations. CEO turnover negatively affects CSRP in the Chinese context. CEO is the leading player in corporate affairs, and his departure causes problems for the firm to sustain its operations. This study's findings provide managers with helpful information for improving organizational performance while also taking into account executives' traits as factors that motivate businesses to report on CSR. Policies should be devised to restrict the executives' turnover and dual roles, while executives with specific attributes, such as CFO, should be given preference.

This study, along with policy implications, has some limitations. First, CSR performance is a significant source of a harmonious society in China. Second, the results reflect one country and attribute a single country's political system. Third, a comparative study may be carried out to look at the variations of CFP, CSRP, ET, CEO-duality, and CEO-CFO nexus between different institutions. Fourth, various aspects of CSRP include a disclosure about the customer and lower staff protections that can be examined in future research.

## Data Availability Statement

The original contributions presented in the study are included in the article/supplementary materials, further inquiries can be directed to the corresponding author.

## Author Contributions

RZ: conceptualization, methodology, formal analysis, writing, and editing. WK: data curation, formal analysis, writing, review, and editing. MK: review, editing, resources, and supervision. All authors contributed to the article and approved the submitted version.

## Funding

This research was supported by the 2022 Scientific Research Fund of Yunnan Province education department (2022J1245).

## Conflict of Interest

The authors declare that the research was conducted in the absence of any commercial or financial relationships that could be construed as a potential conflict of interest.

## Publisher's Note

All claims expressed in this article are solely those of the authors and do not necessarily represent those of their affiliated organizations, or those of the publisher, the editors and the reviewers. Any product that may be evaluated in this article, or claim that may be made by its manufacturer, is not guaranteed or endorsed by the publisher.
